# Laser induced forward transfer imaging using deep learning

**DOI:** 10.1007/s42452-025-06679-x

**Published:** 2025-03-22

**Authors:** James A. Grant-Jacob, Michalis N. Zervas, Ben Mills

**Affiliations:** University of Southampton, Southampton, UK

**Keywords:** LIFT, Laser induced forward transfer, Deep learning, 3D printing, Metal printing, Copper printing

## Abstract

A novel approach for improving the accuracy and efficiency of laser-induced forward transfer (LIFT), through the application of deep learning techniques is presented. By training a neural network on a dataset of images of donor and receiver substrates, the appearance of copper droplets deposited onto the receiver was predicted directly from images of the donor. The results of droplet image prediction using LIFT gave an average RMSE of 9.63 compared with the experimental images, with the SSIM ranging from 0.75 to 0.83, reflecting reliable structural similarity across predictions. These findings underscore the model's predictive potential while identifying opportunities for refinement in minimising error. This approach has the potential to transform parameter optimisation for LIFT, as it enables the visualization of the deposited material without the time-consuming requirement of removing the donor from the setup to allow inspection of the receiver. This work therefore represents an important step forward in the development of LIFT as an additive manufacturing technology to create complex 3D structures on the microscale.

## Article Highlights

- Images of donor surface and receiver surface following laser induced forward transfer (LIFT) are collected.
- A neural network predicts images of deposited copper droplets with high structural accuracy.
- The technique demonstrates non-invasive visualization suitable for real-time imaging of LIFT deposition.

## Introduction

Laser-induced forward transfer (LIFT) is a laser-based printing technique that uses focussed laser pulses to transfer material from a thin-film donor, coated on a transparent substrate, onto a target substrate, known as the receiver [1]. Although backward deposition can also occur [2], LIFT allows for higher resolution deposition using a wide variety of materials. Whilst the mechanism of transfer depends on the laser fluence and materials used, in general, the laser pulse passes through the transparent substrate and is absorbed by the thin-film donor material [3], and the localised expansion results in a region of the material being removed from the donor at high speed and transferred onto the receiver substrate. The applications of LIFT include the printing of electronics [4], sensor applications [5] and functional inks [6]. Whilst high resolution printing can be achieved using electrohydrodynamic inkjet [7], such a technique is limited to viscous material, and others that combine inkjet with selective laser sintering [8] lacking the functionality of LIFT, which excels at controlled deposition of multilayered micron-scale structures without cross-contamination, enabling complex 3D structures or heterogeneous materials.

LIFT has been demonstrated for printing a wide range of materials, including metals [9], conducting polymers [10], graphene [11], and biological substances such as DNA [12], and has been shown capable of producing complex structures, such as continuous copper wires [13], congruent voxels [14], and shaped 2D [15] and 3D structures [16]. However, whilst LIFT has been demonstrated for a huge variety of materials and applications, there has been limited integration of this technique within manufacturing companies, a consequence of the highly sensitive nature of this approach, where there may only be very narrow parameter windows that result in successful deposition. In practice, to optimise a LIFT application, the user will systematically explore the parameter space (e.g. laser fluence), and after each trial the donor is removed in order to image the receiver (as the donor material is generally opaque). This optimisation is hugely time-consuming, and is a major bottleneck because each donor material has distinct physical and chemical properties that affect the transfer process[17], necessitating the fine-tuning of parameters such as laser fluence, pulse duration, and spot size for each new donor to achieve precise and efficient material transfer [18]. Additionally, surface topology and roughness can significantly influence the transfer efficiency and quality, adding another layer of complexity to the optimization process [19]. Multiple experimental iterations are often necessary to identify the optimal conditions, which can be a lengthy process.

Convolutional neural networks (CNNs) [20] can classify objects based on an image input. An extension of the CNN is the conditional generative adversarial network (cGAN), which can transform an image from one domain to another image domain [21], such as from a sketch to a photograph [22], one magnetic resonance image modality to another [23], and has been used in inverse design in photonics [24]. Here we use a cGAN to transform an image of the donor substrate into an image of the receiver substrate, and hence enable the prediction of the appearance of the deposited material directly from an image of the donor. We demonstrate the ability to use neural networks to predict the deposited droplets from LIFT without the need for removing the sample from the setup for microscope imaging. This demonstrates the potential for improving the reliability, capability and accuracy of the deposition process and thus enable more complex 2D and 3D structures. Using deep learning to visualize LIFT deposition (in this case droplets) without removing the sample from the setup is crucial for enhancing efficiency, precision, and cost-effectiveness. It enables non-destructive, real-time monitoring of droplet formation and placement, allowing for instant adjustments to optimise the process whilst preserving sample integrity. This approach eliminates the need for external imaging, reducing downtime and costs, and ensures consistent droplet quality for high-precision applications like microelectronics or biological material transfers. The ability to visualise the deposited sample could allow accurate manufacturing of complex 3D structures on the microscale, such 3D integrated circuits or microelectromechanical systems (MEMS).

## Experiment and method

### Sample fabrication

**Fig. 1:**
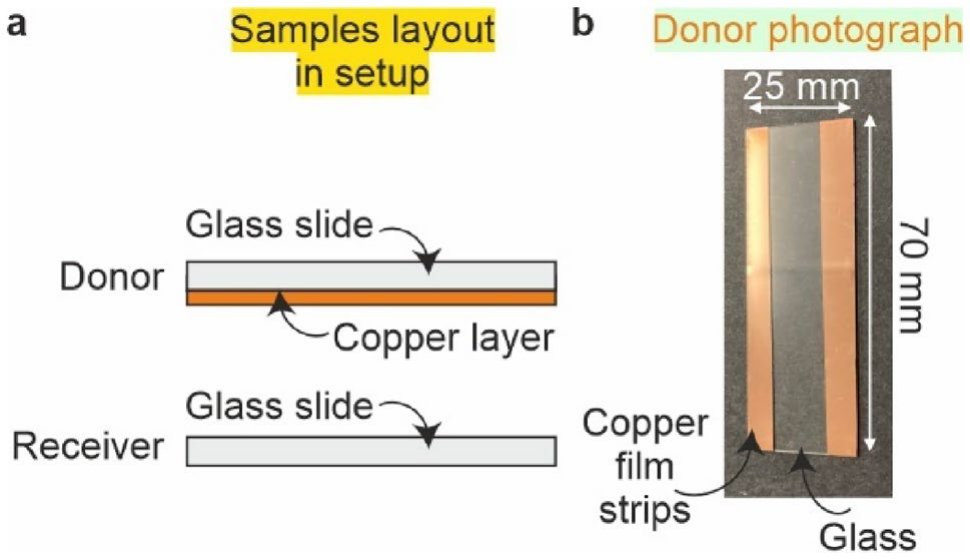
**a** Side view of receiver and donor samples. **b** Photograph of donor copper film with blank region where tape was used to hold sample in deposition chamber

An Edwards Auto 306 thermal evaporator was used to deposit copper onto a glass microscope slide. The slide was 2.5 cm × 7.5 cm × 0.1 cm in size and was attached to the deposition plate using Kapton tape placed along the centre of the slide, hence two regions (strips) were created of copper as shown in the photograph inset in Fig. 1. A chamber pressure of 8 × 10^–6^ mbar was used along with a current of 2.2 A to give a deposition rate of 0.2 nms^−1^, resulting in a 140 nm thick copper film. The receiver was an uncoated glass microscope slide of the same size.

### LIFT setup and data collection

Laser pulses with energy ranging from 0.2 mJ to 1 mJ, with a central wavelength of 1030 nm wavelength and temporal length ~ 190 fs from a Pharos SP Light Conversion ultrafast laser system (see Table 1 for details), that operated in single pulse mode, were focussed onto the surface of the donor copper layer using a 50 × microscope objective (Mitutoyo, NIR, N.A. = 0.42) to give a maximum fluence of 9.8 Jcm^–2^.

**Table 1:** Laser specifications

Specification	Details
Wavelength	1030 nm
Pulse Length	~ 190 fs
Laser System	Light Conversion, Pharos SP
Operating Mode	Single pulse mode
Pulse Energy Range	0.2 mJ to 1 mJ
Focussing Objective	50 × microscope objective (Mitutoyo, NIR, N.A. = 0.42)
Maximum Fluence	9.8 Jcm^−2^

The formula for calculating laser fluence *F* is:

$$F=\frac{E}{A}$$

where:

*F* is the fluence (energy per unit area, Jcm^−2^), *E* is the energy per pulse, measured in joules (J) and *A* is the area over which the energy is distributed. The power of the pulse was determined using a Thorlabs Power S350C meter, whilst the spot size was measured from the ablated region using the microscope images for maximum ablation at 9.8 Jcm^–2^.

**Fig. 2:**
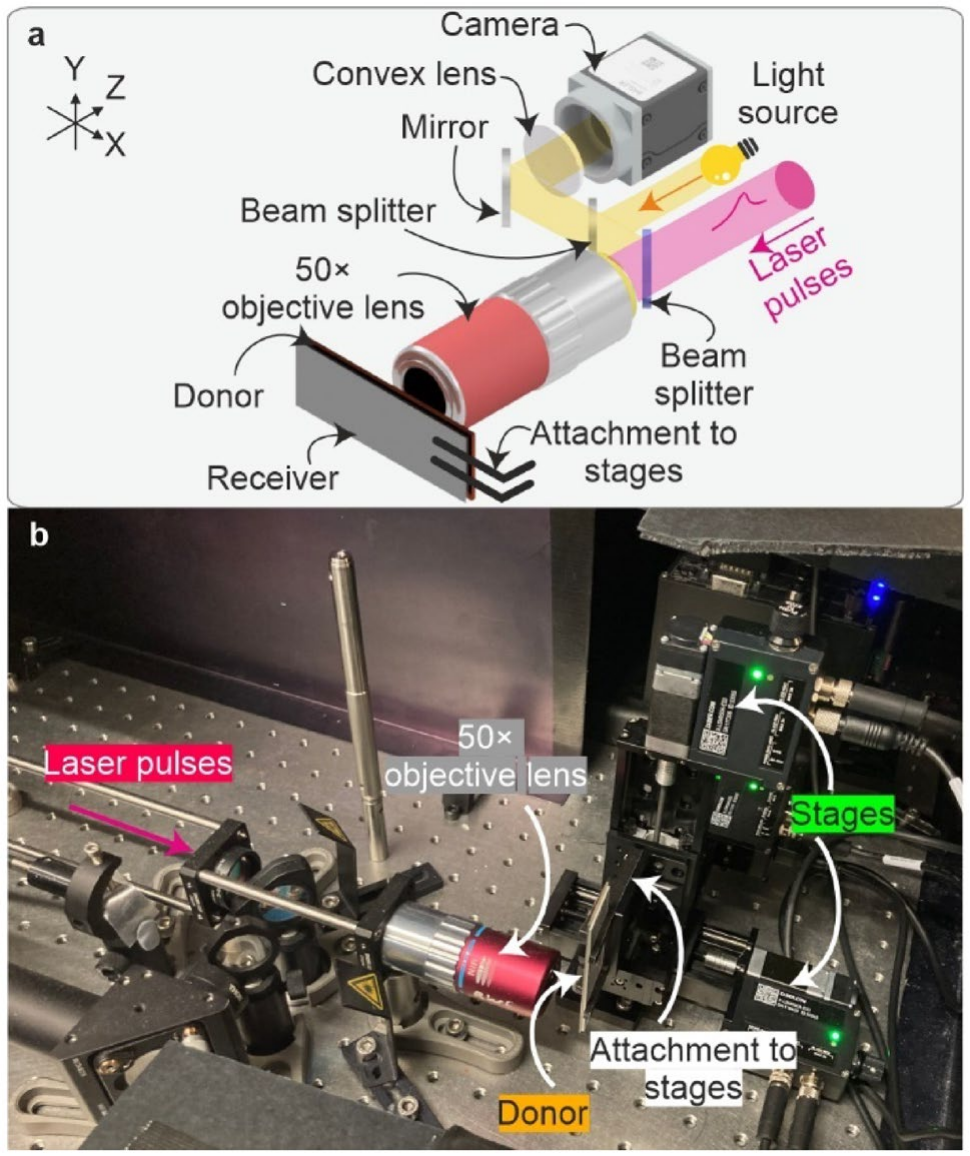
**a** Laser pulses were focussed onto the surface of the donor substrate and copper was deposited onto a receiver. Following depositions, the samples were imaged using a separate Nikon microscope. **b** Photograph of the setup with labels indicating key apparatus

Different pulse energies allowed the forward transfer of copper droplets in a range of different sizes. The objective also allowed the surface to be monitored using a Basler a2A4504-18ucBAS camera (4504 × 4504 pixels, see Table 2) for monitoring LIFT (Fig. 1). The sample was attached via a clamp to a motorised 3-axis stage (Zaber, X-LSM050A-E03) shown in the photograph in Fig. 2b) to allow accurate translation of the sample in the path of the laser pulse, with single pulses directed at the donor surface every 20 µm in XY. Following LIFT, the donor and the receiver were removed from the setup and imaged using a Nikon Eclipse microscope, with a 50 × magnification objective (Nikon LU Plan 0.55 NA, 10.1 mm working distance) and a Basler acA3088-57uc camera (3088 × 2064 pixels, RGB) to collect the training and testing data for the neural network.

**Table 2:** Microscope Basler camera specifications

Sensor Format	Resolution (H x V)	Sensor	Shutter Type	Pixel Size (H x V)
1/1.8"	3088 × 2064	Sony IMX178LQJ-C CMOS	Rolling shutter	2.4 × 2.4 µm

### Neural network

The cGAN (pix2pix variant) [21], was trained for 80 epochs using a Windows 10 computer workstation with two NVIDIA RTX A6000 (48 GB VRAM each). Images of the donor were used as the input and images of the receiver were used as the output, and 400 pairs of images were used to train the neural network. The input and output image pairs for the neural network were cropped from 3088 × 2064 (RGB) to 286 × 286 pixels and resized to 256 × 256 pixels (RGB), and a learning rate of 0.0001 and minibatch size of 2 were used for training. Random augmentation was disabled. The generator depth was set to 7, with 3 input channels and 3 output channels. Finally, the verbose frequency was set to 1000 to control the frequency of training progress updates. ADAM (adaptive moment estimation) [25] was used as the optimiser. Note that the scale bars are not present in the training data for the neural network.

**Fig. 3:**
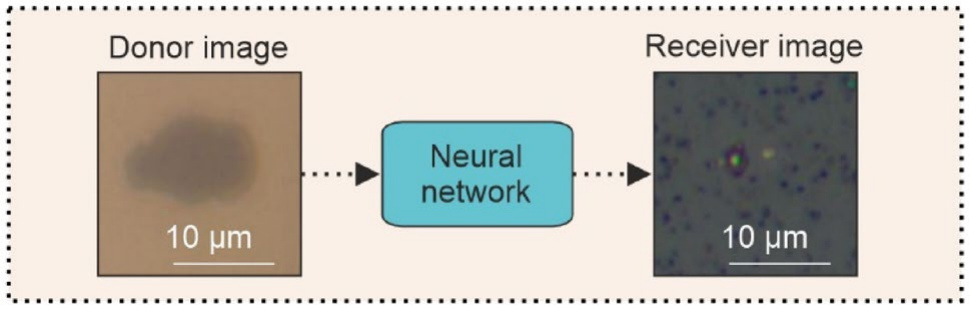
Schematic concept of feeding the donor image into the neural network to produce an image of the receiver

The donor image (as shown in Fig. 3) is an image of the surface of the copper through the glass, i.e., the first copper surface that the laser pulse sees during ablation. Likewise, the receiver image is an image of the surface of the glass slide in which the droplet has been deposited, as viewed from the top of the droplet. Since the droplet is beneath the copper donor surface, it is not possible to accurately image the droplet through the copper layer.

Images of the input are known as the “donor image” and images of the droplets on the receiver surface are known as the “receiver image” as shown in example images in Fig. 3. The hypothesis of this work is that the position and shape of the ablated region in the donor image will provide information to the neural network regarding the appearance of the receiver image. The ability of the neural network to reconstruct the image will depend on the training data, and the quality of the input images. The higher the magnification of the images, the more detail is present for the neural network to create a realistic relationship between donor image and receiver image.

**Fig. 4:**
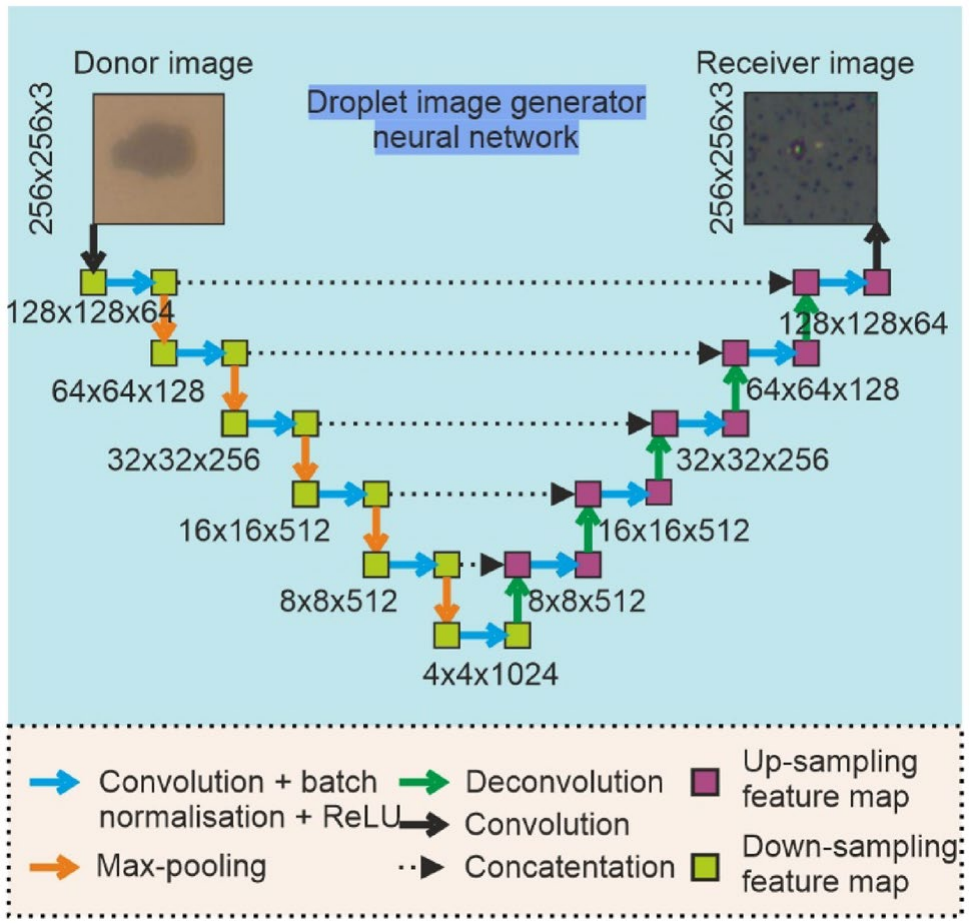
Diagram of the neural network for image generation

A diagram showing how a donor image is fed into a neural network to produce an image of the receiver is shown in Fig. 3, whilst the architecture of the generator network is shown in Fig. 4.

The diagram in Fig. 4 illustrates the U-Net architecture used for droplet image prediction for the LIFT experiment. The model features an encoder-decoder structure, where the encoder extracts spatial features through convolutional layers with batch normalization and ReLU activation, followed by max-pooling to down-sample the spatial dimensions. The decoder reconstructs the receiver image by progressively up-sampling via deconvolutions and concatenating feature maps from the encoder using skip connections. These connections preserve spatial details, ensuring finer structural accuracy in the predicted images. The network processes the donor image (256 × 256 × 3) and outputs a predicted receiver image of the same size, leveraging the U-Net's ability to combine high-level context with detailed spatial information for accurate droplet prediction.

In this work, we used Python (Version 3.8.19, Python Software Foundation, Python Language Reference, available at http://www.python.org) within the Spyder environment (Version 5.5.1, Spyder Project Contributors, Spyder IDE, 64 bit, available at https://www.spyder-ide.org).

**Fig. 5:**
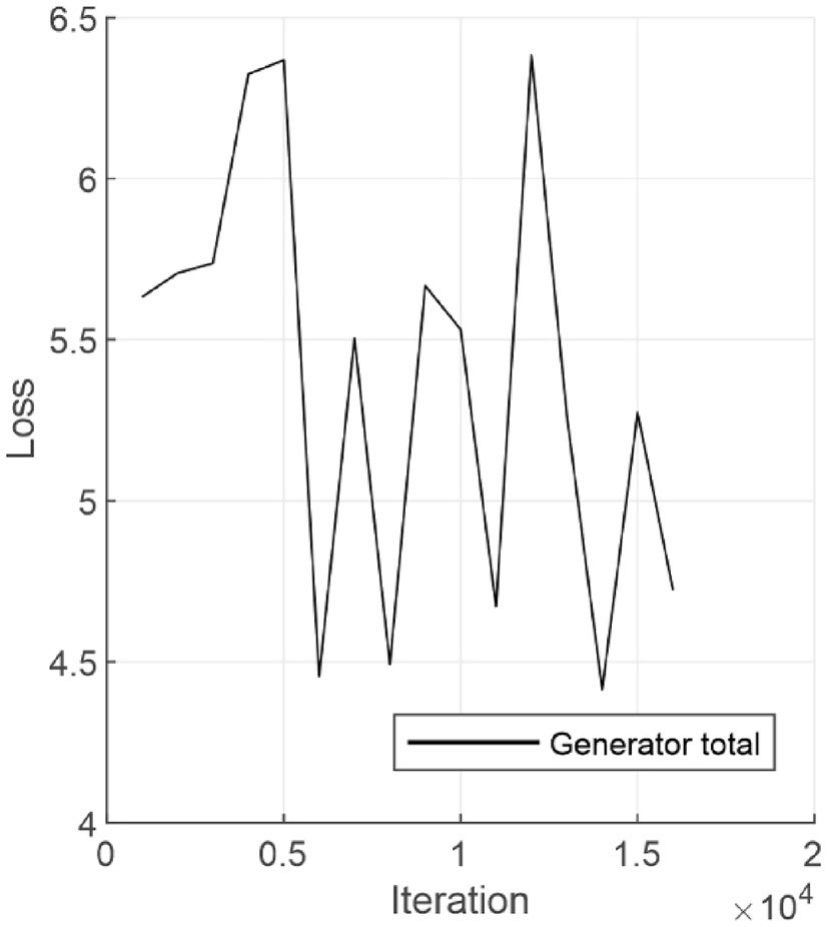
Total generator loss as a function of neural network training iteration

Figure 5 shows the total generator loss during network training, which is the overall error or cost that the generator incurs while trying to generate images that are indistinguishable from the real ones, and the iteration refers to a single update of the model's weights based on a batch of data during training.

**Fig. 6:**
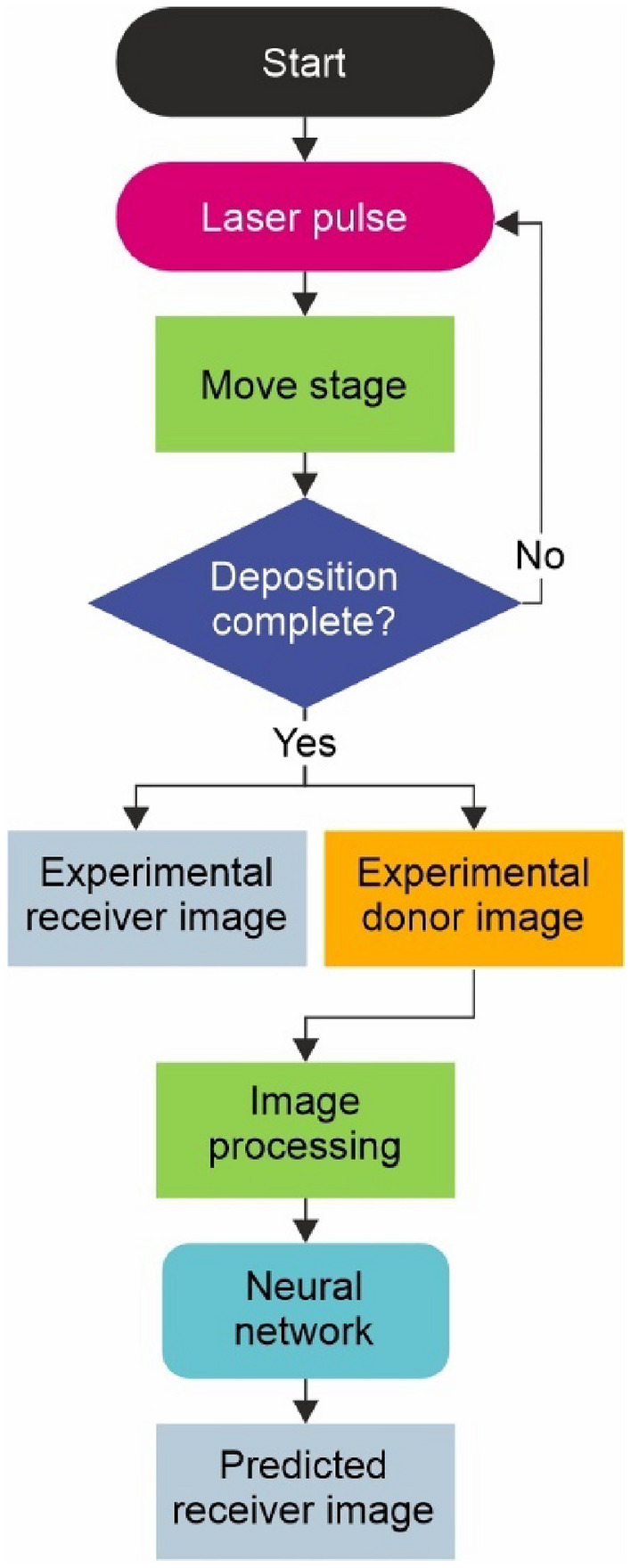
Flowchart of deposition and image prediction process

A flowchart of the process data collection, processing and receiver image prediction is shown in Fig. 6. At the start of the process a laser pulse is fired, the stage is then translated before another pulse is fired and the sequence continues until all depositions are completed. Following this, images of the donor and receiver are obtained using a microscope and are processed (cropped and resized) before the donor image is fed into the neural network for receiver prediction.

## Results and discussion

**Fig. 7:**
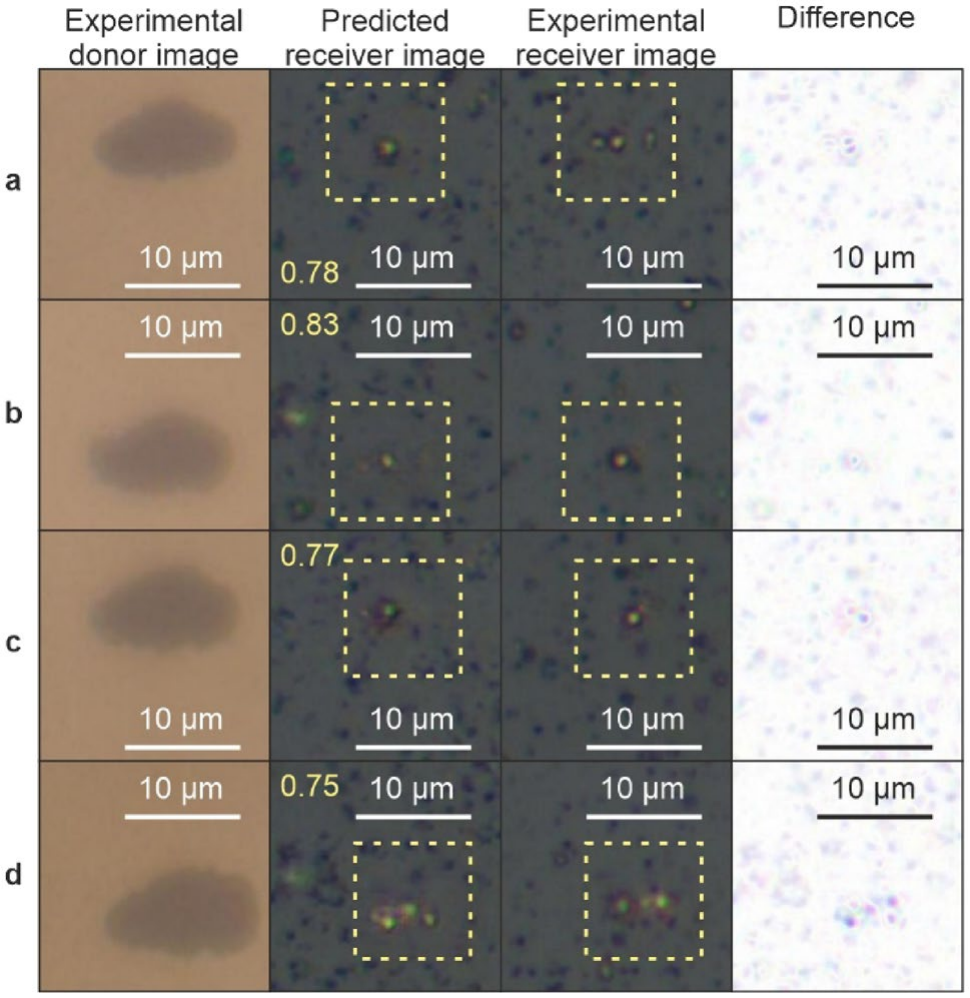
Predicting the appearance of the receiver from images of the donor for laser pulse fluence of **a** 5.4 Jcm^−2^, **b** 6.1 Jcm^−2^, **c** 6.5 Jcm^−2^ and **d** 7.1 Jcm^−2^, with size scales shown in white and SSIM values inset in yellow

Figure 7 shows experimental images of the donor surface (column 1), the neural network predicted images of the receiver (column 2), the associated experimental images of the receiver (the ground truth) (column 3), and the difference between predicted and experimental (column 4), for laser fluences of **a** 5.4 Jcm^−2^, **b** 6.1 Jcm^−2^, **c** 6.5 Jcm^−2^ and **d** 7.1 Jcm^−2^. The neural network predictions of the receiver appear similar in style to the experimental receiver images, and generally show strong agreement in terms of the size and position of the deposited droplets. Single droplets are predicted correctly (**b** and **c**), as are three droplets as shown in (**d)**. The difference column in the figure shows the result of subtracting the predicted image from the experimental, with the darker pixels indicating greater difference between the two sets of images. It is evident the neural network has predicted the colour and the droplet position, but the texture of the background is different. The structural similarity index measurement (SSIM), which is a metric that assesses the resemblance in terms of structure and intensity between two images (where images that are the same give a maximum value of 1), and the root mean squared error (RMSE) were computed for each pair of predicted and experimental receiver images in a 128 × 128-pixel region to assess the predictive accuracy of the network and is shown in yellow.

The SSIM was calculated using the following equation,

$$SSIM(E,G)=\frac{(2{\mu }_{E}{\mu }_{G}+{C}_{1})(2{\sigma }_{EG}+{C}_{2})}{({\mu }_{E}^{2}+{\mu }_{G}^{2}+{C}_{1})({\sigma }_{E}^{2}+{\sigma }_{G}^{2}+{C}_{2})}$$

where *μ*_*G*_ is the mean of *G*, *μ*_*E*_ is the mean of *E*, *σ*_*G*_^2^ is the variance of *G*, *σ*_*E*_^2^ is the variance of *E*, *σ*_*EG*_ is the covariance of *E* and *G*. *C*_1_ = (0.01*L*)^2^ and *C*_2_ = (0.03*L*)^2^, where *L* is the range of the pixel values in the images.

The RMSE, where a lower value indicates greater similarity, was calculated for the predicted receiver images compared to the experimental receiver images. This was done by averaging the squared differences in pixel intensity values (ranging from 0 to 255) between corresponding pixels in the predicted and experimental images, using the following equation,

$$RMSE=\sqrt{\frac{1}{N}{\sum }_{i=1}^{N}{\left({G}_{i}-{E}_{i}\right)}^{2}}$$

where *N* is the number of data points (pixels), *G*_*i*_ is the predicted image pixel value and *E*_*i*_ is the experimental image pixel value. The SSIM and RMSE values computed for the predicted images shown in Fig. 7 are shown in Table 3. The SSIM values for all images exceed 0.75, indicating good structural resemblance between the predicted and experimental images, whilst the RMSE remains below 12 for all images, reflecting low pixel-wise error.

**Table 3:** SSIM and RMSE values for the images shown in Fig. 7

	a	b	c	d
SSIM	0.78	0.83	0.77	0.75
RMSE	8.4	6.9	11.3	11.9

As the neural network was only provided with the experimental image of the donor (and not the laser fluence), this result leads to the important conclusion that information about the deposited material is contained within the donor image. This important result could lead to future work that involves real-time imaging of the deposited material and explores using different materials. Owing to difficulty of creating 3D structures using LIFT, future work should employ the technique demonstrated here with the aim of improving the capability of creating and predicting 3D structures, such as pillars, multi-material structures or MEMS.

The main challenge was making sure that the donor area was correctly aligned to the deposited image area, so that the neural network could successfully understand the transformation. Additional data would be expected to improve the accuracy of image prediction, whilst 3D profiling of the donor ablated surface could also increase the information available to the neural network to predict the deposited droplet.

## Conclusion

To conclude, in this manuscript deep learning has been shown to predict the appearance of deposited material on the receiver, directly from images of the donor. The results of droplet image prediction using LIFT highlight variability in performance across samples. The analysis shows:

• The highest visual accuracy corresponded to the lowest prediction error, with an RMSE of 6.9.
• The poorest performance was observed with the highest RMSE of 11.9, indicating greater deviation between the predicted and experimental images.
• Despite variations in RMSE, the SSIM ranged from 0.75 to 0.83, reflecting reliable structural resemblance between predicted and experimental images.

This application of neural networks in LIFT shows the potential to improve the speed and quality of deposition by unlocking parameter optimisation and deposition visualisation without the time-consuming requirement of needing to remove the donor substrate before the receiver substrate can be imaged, enabling the potential for the fabrication of complex 3D microstructures in real-time.

## Data Availability

Data supporting the results presented in this manuscript are available at https://doi.org/10.5258/SOTON/D3416.
